# End-of-Life Practices in the Intensive Care Unit: The Importance of Geography, Religion, Religious Affiliation, and Culture

**DOI:** 10.5041/RMMJ.10137

**Published:** 2014-01-21

**Authors:** Marc Romain, Charles L. Sprung

**Affiliations:** General Intensive Care Unit, Hadassah-Hebrew University Hospital, Ein Kerem, Jerusalem, Israel

**Keywords:** Attitude, end-of-life care, geography, religion, withdrawing, withholding

## Abstract

End-of-life decisions are made daily in intensive care units worldwide. There are numerous factors affecting these decisions, including geographical location as well as religion and attitudes of caregivers, patients, and families. There is a spectrum of end-of-life care options from full continued care, withholding treatment, withdrawing treatment, and active life-ending procedures.

## INTRODUCTION

**Figure 1.**
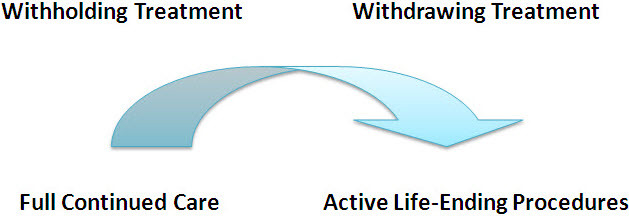
**Spectrum of End-of-Life Decisions.** The figure shows the spectrum of end-of-life decisions from full care to withholding treatment, to withdrawing treatment, to active life-ending procedures.

Medicine has advanced greatly over the last few decades, and as a result patients are living longer. At the same time, physicians in the intensive care unit (ICU) have developed the ability to prolong life even in situations where death is inevitable. Despite these advances, some patients admitted to hospitals will die, and approximately 20% of these patients will die in an ICU.^1^ Of patients who die in American hospitals, approximately half are cared for in an ICU within three days of their death.^2^ In comparison, 10% of patients who die in hospitals in the United Kingdom are cared for in ICUs prior to their death.^3^ This is probably due to fewer available ICU beds.^4^ A few decades ago, patients died in the ICU after undergoing cardiopulmonary resuscitation (CPR).^5^ Today, most patients dying in ICU do so after forgoing life-prolonging therapies.^5^–^8^ Many critically ill patients are initially admitted to the ICU with a prospect of being saved, but when this is not possible a change in the goal to palliative care should occur. This change has been described as moving from cure to comfort.^9^ This change is one of the most difficult decisions faced by intensive care professionals. There is a spectrum of end-of-life care options from full continued care, withholding treatment, withdrawing treatment, and active life-ending procedures (Figure 1). These categories were highlighted in the Ethicus Study.^7^ Full continued care involves all aggressive treatments, including such therapies as mechanical ventilation, vasopressors, and cardiopulmonary resuscitation (CPR).^7^ Withholding treatment was defined as a decision not to start or increase a life-sustaining therapy, for example, not starting a vasopressor or performing CPR. Withdrawing treatment was defined as a decision actively to stop a life-sustaining treatment being given.^7^ Active shortening of the dying process was defined as a circumstance in which someone performed an act with a specific intent of shortening the dying process, for example, giving an intentional overdose of anesthetic or potassium chloride.^7^

## END-OF-LIFE DECISION-MAKING

End-of-life decisions are made daily in hospitals and ICUs around the world. Some common triggers for end-of-life decisions include severe neurological disorders (intraventricular hemorrhage or massive stroke), unresponsiveness to aggressive therapies (continued hypotension despite maximal inotropic support), multi-organ system failure, or irreversible conditions. End-of-life decision-making can be influenced by numerous variables. For example, differences in location (Europe, America, Israel),^6^,^7^,^10^ religious and regional differences,^11^,^12^ and differences amongst attitudes of patients, families, physicians, and nurses.^13^ Wide variations of end-of-life decision-making exist between countries, within countries, within cities, and even within the same ICU.^10^ This can be explained by different physician values. In the United States, medicine has long ago moved away from a paternalistic model to one that promotes autonomy and self-determination.^14^ Patient expectations and wishes are considered regarding end-of-life decisions. In Northern Europe, patient–physician relationships also promote autonomy, but the further south and east you go in Europe, the relationship becomes more paternalistic.^15^,^16^ There is, however, more of a tendency to shared decision-making.^17^ Studies have shown that the majority of physicians in North America and Europe would consider withholding and withdrawing treatment.^6^,^7^ There are still great differences between countries. Doctors in Holland and Belgium perform active euthanasia,^18^,^19^ whereas in Israel physicians withhold but do not usually withdraw treatment.^20^ In fact the withdrawing of ventilators is prohibited by law.^21^

## DIFFERENCES IN GEOGRAPHICAL LOCATION

The Ethicus Study^7^ was a prospective trial performed in European ICUs to determine the frequency and types of actual end-of-life practices. European countries involved were prospectively divided into three geographical regions: Northern (Denmark, Finland, Ireland, the Netherlands, Sweden, and the United Kingdom), Central (Austria, Belgium, Czechia, Germany, and Switzerland), and Southern (Greece, Israel, Italy, Portugal, Spain, and Turkey) Europe. The main outcome variable was the end-of-life category (as defined above). In this study, 31,417 patients were admitted to 37 adult ICUs located in 17 countries over a period of 13.5 months. A total of 4,248 patients (13.5%) who died or had life-sustaining treatments limited in some fashion were included in the study. Limitation of life-sustaining treatment occurred in 3,086 of the 4,248 patients (73%), i.e. in 10% of ICU admissions and 76% of dying patients. Of the 3,086 patients, 2,734 (89%) received mechanical ventilation, and 1,815 (59%) were receiving vasopressors at the first limitation of therapy. There was significant inter-country variability in limitations of care. Twenty percent died with no limitation of therapy and unsuccessful CPR (range 5%–48%), brain death in 8% (range 0%–15%), withholding treatment in 38% (range 16%–70%), withdrawing treatment in 33% (range 5%–69%), and active shortening of the dying process in 2% (range 0%–19%). Of 1,398 patients who underwent withdrawal of treatment, 1,335 (95%) had treatment withheld prior to or together with withdrawing treatment. All patients who underwent shortening of the dying process already had previous treatment withheld or withdrawn.

**Figure 2.**
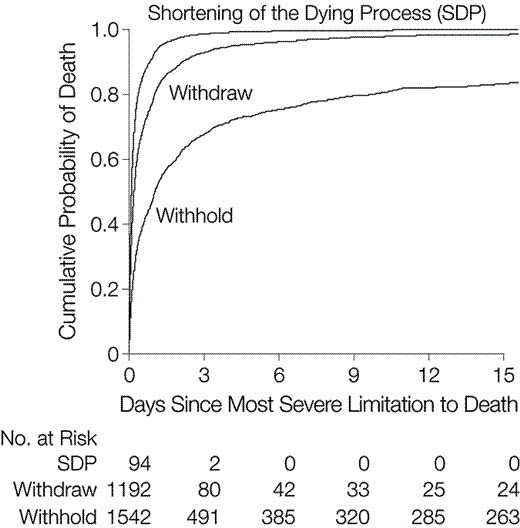
**Probability of Death over Time for Withholding or Withdrawing Treatment or Active Shortening of Dying Process (SDP).** The probability of death is higher and the time to death shorter with SDP than withdrawing and withholding treatment. Adapted with kind permission from JAMA, End of life practices in European intensive care units—the Ethicus Study, Volume 290, 2003, Page 794, Figure, Sprung CL, Cohen SL, Sjokvist P, et al. Ethicus Study Group. Copyright © 2003 American Medical Association. All right reserved.^7^

This study highlights several important points. End-of-life decisions and actions are routine in European ICUs. Withholding and withdrawing treatment seem to be accepted by most European intensivists, while active shortening of the dying process was rare. The study provided useful information for physicians and families regarding approximate times to death after various limitations. For example, death occurred a median of 3.5 (1.5–8.5) hours for shortening of the dying process, 4 (1.0–17.2) hours after withdrawing of therapy, and 14.3 (2.2–67.1) hours after withholding therapy.^7^ The study showed that respective probabilities of death within 24, 48, and 72 hours were 93%, 97%, and 99% for shortening of the dying process, 80%, 89%, and 93% for withdrawing, and 50%, 61%, and 68% for withholding treatments (Figure 2).^7^

The choice to limit therapy rather than continue life-sustaining therapy was related to age, acute and chronic diagnoses, number of days in ICU, frequency of patient turnover, religion, and physician religion. The Northern region had more limitations, decreased CPR, less time until limitation of treatment, and shorter ICU stays.^7^

## ATTITUDES OF PATIENTS, FAMILIES, PHYSICIANS, AND NURSES

Communication between patients (where possible), families, and caregivers of patients in ICU is vital and becomes even more important when considering end-of-life decisions. Another Ethicus paper examined this aspect of end-of-life decisions in European ICUs. Cohen et al.^16^ found that 95% of patients lacked decision-making capacity at the time of the end-of-life decisions. Patients’ wishes were only known in 20% of cases.^16^ End-of-life decisions were only discussed with 68% of families.^16^ Physicians in the Northern countries reported having more information about patients’ wishes (31%) than physicians in Central (16%) or Southern countries (13%). The physicians in Northern countries also had more discussions with families (88%) than Central (70%) and Southern country physicians (48%).^16^ Cohen et al. also found that families were informed 88% of the time about the end-of-life decisions and were only asked about end-of-life wishes 38% of the time.^16^ Reasons for not discussing the end-of-life care with families included the fact that the patient was unresponsive to maximal therapy (39%), the family was unavailable (28%), or it was presumed that the family would not understand (25%).^16^

In the ETHICATT study,^13^ attitudes of Europeans to end-of-life decisions were evaluated. Questionnaires were distributed to physicians and nurses in ICU, patients who survived ICU, and families of ICU patients in six European countries (including Israel). Attitudes regarding quality and value of life, ICU treatments, active euthanasia, and place of treatment were compared. All respondents considered quality of life more important than value of life.^13^ For physicians and nurses, quality of life was more important in end-of-life decisions for themselves than for patients and family. Health professionals, if diagnosed with a terminal illness, wanted fewer ICU admissions, use of CPR, and ventilators (21%, 8%, 10%) than patients and families (58%, 49%, 44%). If faced with a terminal illness with only a short time to live, more physicians (79%) and nurses (61%) than patients (58%) and families (48%) preferred to be at home or in a hospice as opposed to being admitted in a hospital or ICU in order to undergo treatments.

## RELIGION

Religion plays an important role in health, sickness, and death and may also influence end-of-life discussions and decisions.^22^ The Ethicus group subsequently reported on the importance of religious affiliations and culture on end-of-life decisions in European ICUs.^11^ Of the 3,086 physicians surveyed, 1,098 (36%) of the physicians were Catholic, 770 (25%) were Protestant, 669 (22%) had no religion, 309 (10%) were Jewish, 168 (5%) were Greek Orthodox, and 24 (0%) were Moslem. The Ethicus study demonstrated that withdrawal of therapy occurred more frequently for physicians who were Catholic (53%), Protestant (49%), or had no religious affiliation (47%). Withholding of care was more likely to occur than withdrawing if the physician was Jewish (81%), Greek Orthodox (78%), or Moslem (63%).^11^

Religious affiliation also affected the median time from ICU admission to first limitation of care. The median time to overall first limitation of care was 3.2 days but varied according to the physician’s religious affiliations. Greek Orthodox physicians first initiated or limited end-of-life treatment after a median of 7.6 days, Jewish physicians 3.6 days, and Protestant physicians after only 1.6 days.^11^

Religion also affects the decision to discuss the information with the patient’s family. Decisions to limit treatment were discussed with families 68% of the time.^11^ Eighty percent of Protestant physicians, 70% of Catholic physicians, 63% of Jewish physicians, and 55% of Greek Orthodox physicians discussed the decision with the family (*P* < 0.001).^11^

The Catholic Church allows withdrawal of therapy and alleviation of pain and suffering in the dying process, even if life is shortened as an unintentional side effect.^12^,^23^ The principle of “double effect” permits acting when an otherwise legitimate act may also cause an effect one would normally avoid, such as alleviating pain even if it unintentionally hastens death.^12^ The majority of Protestant churches would accept withholding and withdrawing treatments if found appropriate by the treating physician, but there are controversies amongst the Church.^24^,^25^ The Greek Orthodox Church adamantly rejects intentional shortening of life by withdrawing therapy^26^,^27^ and would only allow alleviation of pain if it in no way leads to the patient’s death.^12^ In Jewish law hastening of death is forbidden.^21^,^28^ This is because Jewish law maintains that human life is of infinite value and as a result, withdrawing of life-sustaining treatments is not allowed. It is not only the ends that are important but also the means to that end. For Moslems, withholding and withdrawing therapy are allowed in the terminally ill, but the intention cannot be to hasten death, rather to limit overzealous treatments.^29^ Bulow et al.^12^ summarized the world’s major religions’ points of view on end-of-life decisions (Table 1).

**Table 1.** The Various Religions’ Views on End-of-Life Decisions.

	Withhold	Withdraw	Double Effect ^a^	Euthanasia
Catholics	Yes	Yes	Yes	No
Protestants	Yes	Yes	Yes	Some
Greek Orthodox	No	No	No^b^	No
Moslems	Yes	Yes	Yes	No
Orthodox Jews	Yes	No	Yes	No

a Double effect: Alleviation of pain is allowed, even if it unintentionally hastens death.

b Alleviation of pain is allowed, if it will in no way lead to the patient’s death.

Adapted with kind permission from Springer Science + Business media: Intensive Care Med, The world’s major religions’ points of view on end-of-life decisions in the intensive care unit, Volume 34, 2008, Page 424, Bulow HH, Sprung CL, Reinhart K, et al. Table 1, © Springer-Verlag, 2007.^12^

It is important to point out the interaction between geography and religion. A religious physician’s ethnic beliefs may be tempered by the beliefs of the host society by the process of acculturation.^30^ An example of possible cultural influences can be seen in the way Jewish physicians practice end-of-life decisions. In the Ethicus study, Jewish physicians withdrew in 36% of cases in Northern countries and only 6% in the South.^11^ It is impossible to dissect out the differences between religion and culture as many religions were found in a specific geographical area, such as more Catholic physicians in the Southern countries. This effect has also been seen in America where one study showed that Jewish physicians in Pennsylvania were less likely to withdraw support^31^ as compared to North American Jewish health care providers who were more willing to limit therapy.^32^

## RELIGIOSITY

Bulow et al.^22^ investigated the significant differences in end-of-life decisions between doctors, nurses, patients, and families who consider themselves actively religious and those who identify themselves as only affiliated to a religion. Physicians and nurses wanted less treatment (ICU admission, CPR, ventilation) than patients and family members.^22^ Religious respondents requested more treatment and were more in favor of prolonging life.^22^ Religious respondents were less likely to want euthanasia than those only affiliated to a religion.^22^

Fervent belief in religion usually provides support for families and staff but may lead to significant conflict between staff and parents regarding end-of-life decisions. Brierley et al.^33^ reviewed end-of-life decisions in a pediatric intensive care unit. Of 203 cases in which withdrawal or limitation of treatment was recommended, agreement with family was achieved in 186 (92%). In 17 cases (8%), despite extensive discussions with medical teams and local support mechanisms, no agreement could be obtained. In 11 of these cases (65%), the family expressed explicit religious belief that divine intervention would provide a miracle cure and the medical predictions were wrong.^33^

## OTHER FACTORS

Azoulay et al.^34^ investigated end-of-life practices in 282 intensive care units in seven geographic areas around the world. Of 14,488 patients with available data, 92% did not have decisions to forgo life-saving treatments, and 8% did. Of the 1,239 patients with decisions to limit therapies, 677 (55%) had treatment withheld, and 562 (45%) had treatment withdrawn. As expected, limitations were made in the sickest ICU patients.^34^ Organizational factors seemed to play a role in limitations. For example, patients admitted from another hospital were more likely to have limitations. The presence of a full-time intensivist and availability of doctors on weekends decreased the limitations. Other factors influencing decisions were personal physician characteristics, experience, and gender, case-mix in the ICU, and co-morbidities of patients.^34^

## SUMMARY

End-of-life decisions occur daily in ICUs around the world. There are numerous factors affecting these decisions including geographical location,^6^,^7^,^10^ religion,^11^,^12^ and attitudes of caregivers, patients, and families.^13^ Limitation of therapy appears similar in North American and Northern European ICUs; however, communication, decision-making, and documentation differ in Northern and Southern Europe. End-of-life decisions are different for physicians of different religions.^11^ More withdrawal of care is performed by Catholic, Protestant, or physicians with no religions. Greek Orthodox, Jewish, and Moslem doctors do not withdraw treatment and usually withhold treatment. Greek Orthodox and Moslem doctors are less likely to discuss end-of-life decisions with patients and family. Acculturation may explain why doctors of the same religion have different practices in different locations.^30^ A person’s religion is important, but equally important is whether the person considers themselves religious or not.^22^ Religious respondents wanted more extensive treatment than respondents only affiliated with the same religion. Fewer religious respondents wanted active euthanasia if terminally ill. Patients and families desire more aggressive treatments than doctors and nurses.^13^ Health care professionals must take into account religious and cultural aspects when making end-of-life decisions. When faced with end-of-life decisions, it is important to remember always that while therapies may be withheld or withdrawn, care continues until the very end.

## Abbreviations:

CPR: cardiopulmonary resuscitation;
ICU: intensive care unit;
SDP: shortening of dying process.
